# Voice-Related Quality of Life in Post-Laryngectomy Rehabilitation: Tracheoesophageal Fistula’s Wellness

**DOI:** 10.3390/ijerph17124605

**Published:** 2020-06-26

**Authors:** Salvatore Cocuzza, Antonino Maniaci, Calogero Grillo, Salvatore Ferlito, Giacomo Spinato, Salvatore Coco, Federico Merlino, Giovanna Stilo, Giovanni Paolo Santoro, Giannicola Iannella, Claudio Vicini, Ignazio La Mantia

**Affiliations:** 1 Department of Medical and Surgical Sciences and Advanced Technologies “GF Ingrassia”, ENT Section, University of Catania, 95100 Catania, Italy; s.cocuzza@unict.it (S.C.); grillo@unict.it (C.G.); ferlito@unict.it (S.F.); salvo.coco93@gmail.com (S.C.); federico.merlino93@gmail.com (F.M.); giovastilo@libero.it (G.S.); igolama@gmail.com (I.L.M.); 2 Section of Otorhinolaryngology, University of Padova, 31100 Treviso, Italy; giacomo.spinato@unipd.it; 3 Head and Neck and Robotic Surgery, Department of Experimental and Clinical Medicine, University of Florence, 50134 Florence, Italy; gp.santoroo@gmail.com; 4 Head and Neck Department, ENT & Oral Surgery Unit, G.B. Morgagni, L. Pierantoni Hospital, 47121 Forlì, Italy; giannicola.iannella@uniroma1.it (G.I.); claudio@claudiovicini.com (C.V.); 5 Department of Organi di Senso, University “Sapienza”, 00185 Rome, Italy; 6 Department of Otolaryngology, Head and Neck Surgery, University of Ferrara, 44121 Ferrara, Italy

**Keywords:** quality of life assessment, tracheoesophageal speech, tracheo-esophageal puncture

## Abstract

(1) Introduction: Laryngeal cancer is one of the most common types of cancer affecting the upper aerodigestive tract. Despite ensuring good oncological outcome in many locoregionally advanced cases, total laryngectomy is associated with relevant physical and psychological sequelae. Treatment through tracheo-esophageal speech, if promising, can lead to very variable outcomes. Not all laryngectomee patients with vocal prosthesis benefit from the same level of rehabilitation mainly due to the development of prosthetic or fistula related problems. The relating sequelae in some cases are even more decisive in the patient quality of life, having a higher impact than communicational or verbal skills. (2) Material and Methods: A retrospective study was conducted on 63 patients initially enrolled with a history of total laryngectomy and voice rehabilitation, treated at the University Hospital of Catania from 1 January 2010 to 31 December 2018. Quality of life (QoL) evaluation through validated self-administrated questionnaires was performed. (3) Results: The Voice-Related Quality of Life questionnaire revealed significantly better outcomes in both socio-emotional and functional domains of the tracheoesophageal patient group compared to the esophageal group (*p* = 0.01; *p* = 0.01, respectively), whereas in the Voice Handicap Index assessment, statistically significant scores were not achieved (*p* = 0.33). (4) Discussion: The significant differences reported through the V-RQOL and Voice Handicap Index scales in the presence of fistula related problems and device lifetime reduction when compared to the oesophageal speech group have demonstrated, as supported by the literature, a crucial role in the rehabilitative prognosis. (5) Conclusions: The criteria of low resistance to airflow, optimal tracheoesophageal retention, prolonged device life, simple patient maintenance, and comfortable outpatient surgery are the reference standard for obtaining good QoL results, especially over time. Furthermore, the correct phenotyping of the patient based on the main outcomes achieved at clinical follow-up guarantees the primary objective of the identification of a better quality of life.

## 1. Introduction

Laryngeal cancer is one of the most common types of cancer affecting the upper aerodigestive tract, and accounts for about 177,000 new cases per year worldwide with an estimated 94,800 cancer deaths annually [1,2]. These patients who undergo head and neck surgery often experience challenges in communication, which may impact the quality of life (QoL) [2,3,4]. The World Health Organization (WHO) defines health as “*A state of complete physical, mental, and social well-being not merely the absence of disease*” and the QoL as an “*individual*’*s perception of their position in life in the context of the culture and value systems in which they live and concerning their goals, expectations, standards and concerns*” [5].

Despite ensuring good oncological outcome in many locoregionally advanced cases, total laryngectomy (TL) is associated with relevant physical and psychological sequelae, affecting essential life functions such as breathing, swallowing, and oral communication. Permanent tracheostomy and loss of natural voice worsen patients’ QoL, resulting in social stigma and consequent psychological discomfort.

To date, among the various voice recovery solutions, the tracheo-esophageal puncture (TEP) with the insertion of voice prosthesis (VP) is the method generally recognized as a gold standard procedure for speech restoration after laryngectomy.

Technological advancements have been such that today, indwelling prostheses are designed to meet the criteria of low airflow resistance and optimal retention in the tracheoesophageal party wall, have prolonged device lifetime, simple maintenance by the patient, and comfortable outpatient replacement, with success rates classically from 40 to 90% with excellent voice quality [6,7,8,9]. More recent findings by Souza et al. in a study population of 95 patients reported the overall quality of life in voice prosthesis from good to excellent in 83.2% of cases, with better results compared to esophageal voice [8].

Several studies have previously performed an assessment of QoL and degree of satisfaction in TL patients after VP rehabilitation, demonstrating its good efficacy in highly motivated subjects, even over time [9,10].

Despite this, it is necessary to consider, however, that not all laryngectomee patients with vocal prosthesis benefit from the same level of rehabilitation. Indeed, the spread of known prosthetic device lifetime or fistula related problems, which is a more or less decisive way, has an impact on the level and the quality of speech rehabilitation. A drastically reduced device lifetime, periprosthetic leakage, recurrent tracheoesophageal granulomas, and poor vocal performance forces the patient to undergo a higher number of medical-surgical procedures, often aggressive, with potential secondary psychophysical discomfort. The following rehabilitation level often results in not being optimal and autonomous, leading to a possible failure and closure of the phonatory fistula. To the above, numerous reports have identified pathological or uncontrolled gastroesophageal reflux, the chronic mucosal outcomes of radiotherapy, as known etiopathogenetic factors that, alone or in association, have a decisive impact on voice prosthesis outcomes [11].

Therefore, the presence of good communication indices is not necessarily related to the degree of satisfaction of the prosthetic treatment and the related socio-emotional consequences [12].

To this end, through the use of dedicated evaluation questionnaires within a court of laryngectomies and prosthetic patients, we retrospectively evaluated the variability of the related QoL based on specific indicators. Categorization, according to the presence or absence of prosthetic or fistula-related disorders comparing the obtained results with an esophageal speech group was performed.

## 2. Material and Methods

A retrospective study on the QoL of laryngectomized patients with a different degree of tracheoesophageal voice prosthesis rehabilitation was performed. The results were then compared with a group of patients with esophageal voice rehabilitation.

### 2.1. Selection Process

A total of 63 patients initially treated at the University Hospital of Catania from 1 January 2010 to 31 December 2018 were initially considered.

Patients were enrolled in compliance with the following inclusion/exclusion criteria:

1. Inclusion Criteria: Tracheoesophageal speech utilizing a voice prosthesis after laryngectomy procedures for laryngeal tumor (both primary and secondary TEP performed); esophageal speech; clinical-instrumental follow-up after TEP performed ≥ 10 years.
2. Exclusion Criteria: patients with evident local recurrence of pathology; the presence of comorbidities with significant impact on the patient’s QoL (neurological, cerebrovascular, or cardiovascular accident, myopathy); not related psychological diseases occurred after surgery; exitus for external causa before completing the study.

### 2.2. Patients Population

A total of nine patients were excluded due to the selection criteria while 54 patients were successfully interviewed, and enrolled. The enrolled patients consisted of 47 males and seven females, aged from 53 to 78 years (mean age: 64.7 years). TEP was performed as a primary procedure in 10 cases while as a secondary one in 29 cases, generally almost six months after TL (6.1 ± 1.4 months). Instead, esophageal speech rehabilitation was achieved in 15 patients.

All sociodemographic and clinical characteristics such as the mean age, patient sex, mean follow up, TEP procedures, and the history of radiotherapy treatment are summarized in Table 1.

All data were collected from the same two surgeons (S.C. and I.L.M.) who followed the post-operative control up to its fulfilment.

According to prosthesis outcomes and fistula related disorders observed at clinical follow-up, we classified patients into two patient groups:

1. Group 1 including all patients treated with tracheoesophageal voice rehabilitation.
2. Group 2 defined by patients performing esophageal voice rehabilitation.

Moreover, in the second part of the analysis, based on the main complications recorded in the TEP group, we identified two phenotypic subgroups and assessed specific QoL results for each one:

- Prosthetic disorders group (PD), defined by a prosthetic device lifetime ≤3 months; and
- Fistula-related disorders group (FRD), defined by the presence of subsequent complications such as periprosthetic leakage, macro fistula, recurrent tracheoesophageal granuloma).

### 2.3. Outcome Assessment

Patients who respected the selection criteria underwent subjective and objective evaluations:

1. Quality of life Assessment obtained through the administration of the Voice-Related Quality of Life questionnaire (VR-QoL);
2. Vocal performance evaluation through the Voice Handicap Index (VHI);
3. Percentage of annual complications (failure due to periprosthetic leakage, presence of tracheoesophageal granulation tissue, spontaneous dislodged prosthesis, macro fistula);
4. Median device lifetime duration per year, subsequently divided in each sub-category, through consecutive follow up was recorded.

### 2.4. Quality of Life (QoL) Assessment

This was carried out through the use of a specific questionnaire administered in each of the two main identified groups, the TEP group (total, prosthesis and fistula related disorders) and ES group.

The V-RQoL questionnaire is a self-administered assessment tool that evaluates the subjective burden elicited by a voice disorder [13]. It is made up 10 statements on aspects related to voice through the emotion, physical, and functional domains with a score of 0–50. Therefore, the degree of severity of the score is directly proportional to the total numerical sum, detecting the poor quality of life for high total values obtained.

The score has two domains, of which six items evaluate the physical functioning (PF) and four the social-emotional (SE).

### 2.5. Subjective Voice Disorders Assessment

The VHI is made up of three parts concerning the emotional, physical, and functional component, respectively, each composed of 10 items. Each item is marked on a 4-point scale, and the score ranges from 0 to 120, where 120 represents the maximum perceived disability. The score resulting is divided into mild impact (0–40 points), moderate impact (41–60), and severe impact (score > 60).

### 2.6. Statistical Analysis and Ethical Statement

Data analysis was performed using IBM SPSS Statistics for Windows, IBM Corp. Released 2017, Version 25.0. Armonk, NY: IBM Corp. Descriptive statistics were reported on average ± standard deviation or proportion. Data normality was assessed using the Kolmogorov–Smirnov test of normality. The T-test for paired samples was used to determine the difference between observations. The Mann–Whitney U test was performed to analyze group differences. The tests were two-tailed, and a *p*-value of < 0.05 was considered as statistically significant.

The study protocol was approved by the ethics committee of the involved Institution (CE Catania 2; Prot. N. 298/BE). Participants were informed and gave written informed consent of the purpose and procedures of the study, which was conducted according to the Declaration of Helsinki.

## 3. Results

A total number of 54 patients were successfully interviewed after initial selection (47 males and seven females). After the selection process, 39/54 (72.2%) who performed TEP rehabilitation were included, whereas 15/54 (27.8%) among the subjects rehabilitated with ES voice were selected. The ATOS medical puncture set and prosthesis system were used in all patients.

The timing of the TEP procedure performed was primary in 10 (18.5%) patients while secondary in 29 (53.7%) patients.

There were no significant differences between the two groups regarding the demographic and clinical data recorded as summarized in Table 1.

### 3.1. Voice Prosthesis Sequelae and Device Lifetime

At the follow-up conducted in the TEP group, a leakage was detected in 18/39 (46.1%) cases who underwent fistulization. In particular, eight (20.5%) (Group PD) patients presented leakage through the prosthesis, associated with an average device lifetime fewer than 90 days, while 10/39 (25.6%) (Group FRD) patients presented peri prosthesis leakage. In this last group, the related sequelae identified were granulation tissue in 7/39 (17.9%) whereas there were changes in fistula size in 3/39 (7.7%) (Table 2).

The mean device lifetime recorded in TEP patients was 83.53 ± 8.3. Through the TEP group sub-typing based on the detected complications, we found a 3-month lower device lifetime only in the patients with prosthetic disorders (PD) compared to the healthy TEP patients (H) and fistula related (FR) ones, respectively (PD 61.9 ± 9.6 days; H 97.4 ± 8.8 days; FR 91.3 ± 6.5 days) (Table 2).

### 3.2. Voice-Related Quality of Life Questionnaire Assessment

The VrQoL questionnaire revealed significantly better outcomes in both *Socio-Emotional* and *Functional* domains of the TEP patient group compared to the EV group (*p* = 0.01; *p* = 0.01, respectively) (Table 3).

The evaluation of the subgroups divided by specific complications showed better results in the group of prosthetic disorders, despite the reduced prosthesis lifetime, compared to the esophageal speech patients (*p* = 0.002). However, the V-RQoL scoring showed a worsening of the well-being indices in the group with disorders related to the tracheo-esophageal fistula compared to the EV group (*p* < 0.001) (Table 3).

### 3.3. Voice Handicap Index (VHI) Score and Grading

The results of the patient groups analyzed are shown in the VHI score (Table 4). The TEP group showed better total VHI score than the EV group, but was not statistically significant (36.24 ± 7.19 vs. 38.53 ± 6.62; *p* = 0.33). Further examination of the pathological sub-phenotypes presented better scores in the voice prosthesis disorders group than in the esophageal speech (30.37 ± 4.88 vs. 38.53 ± 6.62; *p* = 0.01). However, the outcomes analysis of the patients with fistula disorders showed a worsening of the scores compared to esophageal speech (54.1 ± 10.48 vs. 38.53 ± 6.62; *p* = 0.003) (Table 4).

## 4. Discussion

The benefit of tracheoesophageal voice rehabilitation was formulated first in 1972 by Mozolewski et al. [14]. Since then, many devices have been produced by different companies with variable technologies and specific architecture features, generally taking advantage of a voice polymer prosthesis inserted through a puncture in the shared wall between the trachea and esophagus [11,15,16,17,18,19].

Countless clinical variables such as gastroesophageal reflux, ageing effect, adjuvant radiotherapy, or timing of surgery can influence the laryngectomee patient to failure of vocal rehabilitation treatment and, consequently, the quality of life (QoL) [7,20,21,22,23,24,25].

Precisely, pathological supraesophageal reflux correlates with the onset of fistula complications and the consecutive rehabilitation degree, inducing the onset of periprosthetic leakage [26]. In this regard, Lorenz et al. found higher VHI scores (up to 64.1 ± 9.6) with reflux severity (*p* = 0.025) and total quality of life scores were worse in patients with highly pathological reflux (*p* = 0.007).

Furthermore, the association of post-surgery radiotherapy in patients with voice prosthesis (PORT) can play a determining role in the genesis of gastroesophageal reflux and fistula-related pathology [27]. Cocuzza et al. compared two patient groups based on the choice of treatment and found a significantly higher rate of failure of voice rehabilitation in subjects with gastroesophageal reflux and history of postoperative radiotherapy (45% vs. 17%; *p* < 0.05).

The role of the correct therapeutic choice in the literature is much debated because of the possible consequent rehabilitation complications, making a careful selection of the patients’ candidacy for TEP necessary [6,7,27,28,29,30,31]. For instance, the variable choice between radiotherapy protocols can lead to different long-term rehabilitative outcomes, depending on the therapeutic tissue dosage. As discussed by Elving et al., a dose equal or more than 60 Gray to the primary tumor site limited the prosthesis device lifetime (*p* < 0.05) [31].

The direct consequence consists of the remodeling of the pharyngeal microflora, which under physiological conditions produces mucins that are active against the primary pathogens of the Candida group and successive anomalous prosthetic colonization [32,33,34].

Later, Agarwal et al. discussed the role of treatment modalities including the surgical procedure and details such as neck dissection and pharyngeal reconstruction as well as radiation therapy on quality of life measured by the VHI and V-RQoL questionnaires [35]. Although the surgery did not show significant results, postoperative radiotherapy was initially associated with a higher level of voice handicap. However, the same score during follow-up was significantly decreased, according to the authors due to lessened tissue flexibility in early post-radiotherapy.

Either the disorders related to a reduced prosthetic lifetime or the fistula-related ones previously indicated can influence vocal and communication skills with significant sequelae on the quality of life, inducing the patients to make changes in their behavioral and social sphere [12,36].

The World Health Organization defines the quality of life as ‘’the perception of the individual in his own life, in the context of cultural systems and values, of their objectives, expectations, standards and concerns’’ [5].

Undoubtedly, many determinants may limit tracheoesophageal voice rehabilitation, influencing either the patient’s quality of life or vocal performance, but each of these can change the degree of treatment satisfaction variably. To this end, subjective questionnaires were designed to allow a self-assessment QoL in specific domains of an individual’s life, also introducing the fundamental concept of voice-related quality of life [37,38,39].

Given these assumptions, Moukarbel et al. analyzed the voice-related quality of life (V-RQoL) outcomes specific to tracheoesophageal esophageal speech post-laryngectomy in 75 patients initially enrolled [39].

Although the paired comparison between the TEP and ES group showed a significant difference in socio-emotional and total scores (*p* < 0.05), however, the analysis of physical-functional domains was not significantly different. The data emerging from our analysis confirmed a statistical difference between the overall results of the TEP and EV groups (8.73 ± 4.71 vs. 10.76 ± 2.21; *p* = 0.01). Nevertheless, the observation of the two domains showed a significant result both in the functional *p* = 0.01 than in the socio-emotional one (*p* = 0.01).

Further assessment was performed by Agarwal et al., correlating voice-related QoL and socioeconomic status after total laryngectomy [35]. From the 104 patients who underwent total laryngectomy initially enrolled, only 71 were eligible for the study, administering the V-RQoL, and VHI questionnaires after 1-year of TEP rehabilitation. Long-term outcomes of the V-RQoL reported a higher patient satisfaction in the relation of the crucial contribution of social support (about 80% V-RQoL excellent score and >75% minimal VHI score).

In our study, although the TEP group patients obtained higher results than those in the esophageal speech group, statistical significance was not achieved when comparing VHI scores (36.24 ± 7.19 vs. 38.53 ± 6.62; *p* = 0.33). Therefore, the investigations on the relationship between comprehensibility, intensity, and what voice handicap can entail offers qualitative learning on the experience lived and the social impact of the voice prosthesis.

However, bias may be present in our findings from our non-randomized study regarding surgery type choice conducted, according to non-standardized protocols, and therefore outcomes may not be assessed blind.

Later in 2016, Ţiple et al. described the impact of vocal rehabilitation on quality of life and voice handicap in patients with a total laryngectomy, dealing in particular with the different adaptation timing to the rehabilitation method used [38].

Although the first use of the VHI questionnaire in patients with TEP was initially associated with severe voice handicap than the esophageal group (ES 52.67 ± 19.32 vs. TEP 61.57 ± 24.28); after an adjustment period of six months, the second application of the questionnaires showed a significant improvement in voice production (59.58 ± 16.33).

This assumption explains why patients with TEP need a longer considerable adaptation period to integrate back into society and suit the new conditions of life than esophageal voice.

In the previous study, our patient analysis based on the handicap of the voice after a follow-up of at least 10 years revealed a better, but not significant, TEP score of an EV, respectively (36.24 ± 7.19 vs. 38.53 ± 6.62; *p* = 0.33).

As described in the literature, the specific QoL outcomes of the TEP differ significantly according to the different risk factors that are concentrated within the TEP patients, therefore, the patients with excellent vocal performance, but reduced device lifetime, and patients with fistula related disorders [7,8,9,10,11,39,40]. Nevertheless, to our knowledge, this is the first published analysis of phenotyping different subgroups into tracheoesophageal speech patients.

In our study, a short device lifetime (<3 months) did not result in a limiting factor of tracheoesophageal (TE) voice restoration in both the V-RQoL score and the VHI compared with the EV group (*p* = 0.002; *p* = 0.01, respectively).

However, opposite data were found in the fistula-related disorders group with worse V-RQoL and VHI outcomes than patients with esophageal voice (*p* = 0.0007; *p* = 0.003, respectively).

As discussed above, despite favorable evidence regarding treatment, a percentage of subjects in the follow-up either considered removing the voice prosthesis or said that they would not choose the same type of voice rehabilitation if they could go back in time [10].

### Limitations

The main limitation of the study we performed is the modest number of patients analyzed, which may have resulted in the research being underpowered. However, it is essential to consider that after applying such rigid inclusion and exclusion criteria such as an extended follow-up, the total number of selected patients was drastically reduced, making it a representative sample in any event.

Furthermore, bias may be present in our findings from our non-randomized study regarding surgery type choice conducted according to non-standardized protocols. Therefore, outcomes may not be assessed blind.

## 5. Conclusions

The prosthetic rehabilitation treatment allows for the recovery of the laryngectomee patient’s communication skills, positively affecting both the cognitive-emotional component and the physical-functional one. The variable presence of sequelae acts as a risk factor for the worse quality of life of the patient, and the accurate choice of rehabilitative management represents the primary target. Although the TEP patient has excellent long-term outcomes, the correct phenotyping of complications can provide the keystone for better administration, identifying subjects who could better benefit from alternative rehabilitation procedures.

## Figures and Tables

**Table 1:** Demographic and clinical features.

Characteristic	No. (%)/Range	Mean	SD
Sex			
Male	47 (87%)		
Female	7 (13%)		
Age	53–78 y	64.7 y	±7.58 y
Mean follow up, y		11.2 y	±1.65 y
T Stage			
III	35 (64.8%)		
IV	19 (35.2%)		
Neck dissection			
Yes	39 (72.2%)		
No	15 (27.8%)		
Radiation			
Irradiated	35 (64.8%)		
Not irradiated	19 (35.2%)		
TEP Procedure			
Primary	10 (18.5%)		
Secondary	29 (53.7%)		
Esophageal Voice	15 (27.8%)		

Abbreviations: T stage = tumor stage; TEP = tracheoesophageal prosthesis.

**Table 2:** Percentage of complications per patient.

Complications TEP Group	No. (%) y
Prosthesis leak	
Through	8/39 (20.5%)
Peri	10/39 (25.6%)
Granulation’s tissue	7/39 (17.9%)
Fistula size changes	3/39 (7.7%)
Device lifetime	Mean days (SD)
H group	97.4 ± 8.8 days
FT group	91.3 ± 6.5 days
PD group	61.9 ± 9.6 days

Abbreviations: H = healthy; FT = Fistula type; PD = Prosthesis disorder group.

**Table 3:** Comparison of V-RQoL outcomes.

VrQoL	Tracheoesophageal Voice Prosthesis (TEP)	Voice Prosthesis Disorders (PD)	Fistula Related Disorders (FRD)	Esophageal Speech (EV)
No. Patients	39	8	10	15
Socio-Emotional	4.15 ^a^ ± 2.23	3.47 ± 0.54	7.18 ± 2.22	4.78 ^a^ ± 1.03
Functional	4.57 ^b^ ± 2.48	4.16 ± 1.19	8.33 ± 1.23	5.98 ^b^ ± 1.18
*Total*	8.73 ^c^ ± 4.71	7.63 ± 1.73 ^d^	15.51 ± 3.45 ^e^	10.76 ^c, d, e^ ± 2.21

Abbreviations: VrQoL = Voice Related Quality of Life; TEP = tracheoesophageal prosthesis; No. patients = number of patients. Comparison ^a^ Socio-Emotional TEP vs. EV *p* = 0.01; ^b^ Functional TEP vs. EV *p* = 0.01; ^c^ Total TEP vs. Total EV *p* = 0.01; ^d^ Total PD vs. EV *p* = 0.002; ^e^ Total FRD vs. EV *p* = 0.0007.

**Table 4:** Comparison of each sub-classes voice handicap index (VHI) scores. Statistical significance at *p* < 0.05.

	Tracheoesophageal Voice Prosthesis (TEP)	Voice Prosthesis Disorders (PD)	Fistula Related Disorders (FRD)	Esophageal Speech (EV)
**VHI**				
Emotional	9.59 ± 2.14	8.87 ± 0.99	13.1 ± 3.81	9.4 ± 1.35
Physical	12.12 ± 2.15	10.25 ± 1.58	18.5 ± 3.43	12.53 ± 2.58
Functional	14.53 ± 2.89	11.25 ± 2.31	22.5 ± 3.24	16.6 ± 2.69
Total Score	36.24 ^a^ ± 7.19	30.37 ^b^ ± 4.88	54.1 ^c^ ± 10.48	38.53 ^a, b, c^ ± 6.62

Abbreviations: Voice Related Quality of Life; TEP = tracheoesophageal prosthesis. Comparison: ^(**a**)^ TEP group vs. EV group *p* = 0.33; ^(**b**)^ PD group vs. EV group *p* = 0.01; **^(c)^** FRD vs. EV *p* = 0.003.
